# Regulatory Role and Potential Importance of GDF-8 in Ovarian Reproductive Activity

**DOI:** 10.3389/fendo.2022.878069

**Published:** 2022-05-26

**Authors:** Xiaoling Zheng, Yongquan Zheng, Dongxu Qin, Yao Yao, Xiao Zhang, Yunchun Zhao, Caihong Zheng

**Affiliations:** ^1^ Department of Pharmacy, Women’s Hospital, School of Medicine, Zhejiang University, Hangzhou, China; ^2^ Key Laboratory of Reproductive Genetics (Ministry of Education) and Women’s Reproductive Health Laboratory of Zhejiang Province, Women’s Hospital, Zhejiang University School of Medicine, Hangzhou, China

**Keywords:** GDF-8, PCOS (polycystic ovarian syndrome), ovarian response, pregnancy outcome, granulosa cells (GC), SMAD signaling

## Abstract

Growth differentiation factor-8 (GDF-8) is a member of the transforming growth factor-beta superfamily. Studies *in vitro* and *in vivo* have shown GDF-8 to be involved in the physiology and pathology of ovarian reproductive functions. *In vitro* experiments using a granulosa-cell model have demonstrated steroidogenesis, gonadotrophin responsiveness, glucose metabolism, cell proliferation as well as expression of lysyl oxidase and pentraxin 3 to be regulated by GDF-8 *via* the mothers against decapentaplegic homolog signaling pathway. Clinical data have shown that GDF-8 is expressed widely in the human ovary and has high expression in serum of obese women with polycystic ovary syndrome. GDF-8 expression in serum changes dynamically in patients undergoing controlled ovarian hyperstimulation. GDF-8 expression in serum and follicular fluid is correlated with the ovarian response and pregnancy outcome during *in vitro* fertilization. Blocking the GDF-8 signaling pathway is a potential therapeutic for ovarian hyperstimulation syndrome and ovulation disorders in polycystic ovary syndrome. GDF-8 has a regulatory role and potential importance in ovarian reproductive activity and may be involved in folliculogenesis, ovulation, and early embryo implantation.

## Introduction

The internal structure of the ovary can be divided into cortex and medulla, mainly composed of follicles and connective tissue, containing both germ cells (eggs or oocytes) and somatic cells. The latter include thecal cells (TCs) stromal cells and granulosa cells (GCs). The interactions between germ cells and somatic cells are critical and affect many aspects of ovarian function, such as the formation of oocyte-containing follicles; development of oocytes and somatic cells as follicles; ovulation; formation of the corpus luteum (1). Normal ovarian function is dependent not only on endocrine factors (e.g., hypothalamus secretes gonadotropin-releasing hormone (GnRH) that stimulates the anterior pituitary gland to secrete luteinizing hormone (LH) and follicle-stimulating hormone (FSH)) but also on many locally produced factors that exert their effects in an autocrine manner and/or a paracrine manner. Abnormal expression of locally produced factors can disrupt the balance of the microenvironment and, thus, impair ovarian function (2, 3).

The transforming growth factor-beta (TGF-β) superfamily consists of TGF-βs, activins, bone morphogenetic proteins (BMP), anti-Müllerian hormone (AMH), growth-differentiation factors (GDF), and other proteins. Several publications have documented that local production of BMPs, AMH, GDFs, and activins in the ovary is associated with the formation and function of mammalian germ cells. It has been reported that AMH (4), activin A, activin B, activin AB (5), BMP2 (6), BMP4 (7), BMP6 (8), BMP7 (9), BMP9 (10), BMP15 (11), GDF3 (12), GDF9 (11, 13) and their receptors are expressed in the human ovary. Their tissue-specific and time-dependent expression affects oocyte-somite interactions, steroidogenesis, GC proliferation, oocyte maturation, cumulus expansion, ovulation, embryo mass and luteal function.

Growth differentiation factor-8 (GDF-8), also known as myostatin, is a member of the TGF-β superfamily and is a secreted protein that is synthesized mainly by skeletal muscle cells. Animal experiments have shown that GDF-8 is a negative regulator of skeletal muscle growth (14). Mice lacking the GDF-8 gene increased approximately 25-30% muscle mass due to muscle fiber hyperplasia, compared to wild-type mice (14). Naturally occurring gene mutations of GDF-8 with similar phenotypes have been identified in several species including humans (15–17). In addition, GDF-8 has been shown to be involved in metabolism, including lipid synthesis (18–21), obesity (22, 23), and insulin resistance (IR) (24, 25).

GDF-8 signals mainly through type-I serine/threonine kinase receptors (activin receptor-like kinase 4 (ALK4) or ALK5) and the type-II serine/threonine kinase receptors (activin receptor type 2A (ACVR2A) or ACVR2B) (26–28). The specificity of biological responses is determined primarily by the type-I receptor. After ligand binding, type-I receptors are activated and induce the phosphorylation of mothers against decapentaplegic homolog 2 (SMAD2) and SMAD3. Phosphorylated SMAD2 and SMAD3 bind with SMAD4 to form a complex, which then translocates to the nucleus to initiate distinct downstream signaling cascades (29).

Over the past few years, there has been increasing interest in studying the functional roles of GDF-8 outside the musculoskeletal system. Recent studies have shown that GDF-8 and its receptors are expressed widely in reproductive tissues, such as the uterus (30, 31), placenta (32), and ovary (33). Furthermore, compelling data have revealed GDF-8 involvement in the pathophysiology of several types of reproductive dysfunction, such as uterine myoma (31), preeclampsia (34, 35) and polycystic ovary syndrome (PCOS) (33).

**Scheme 1:**
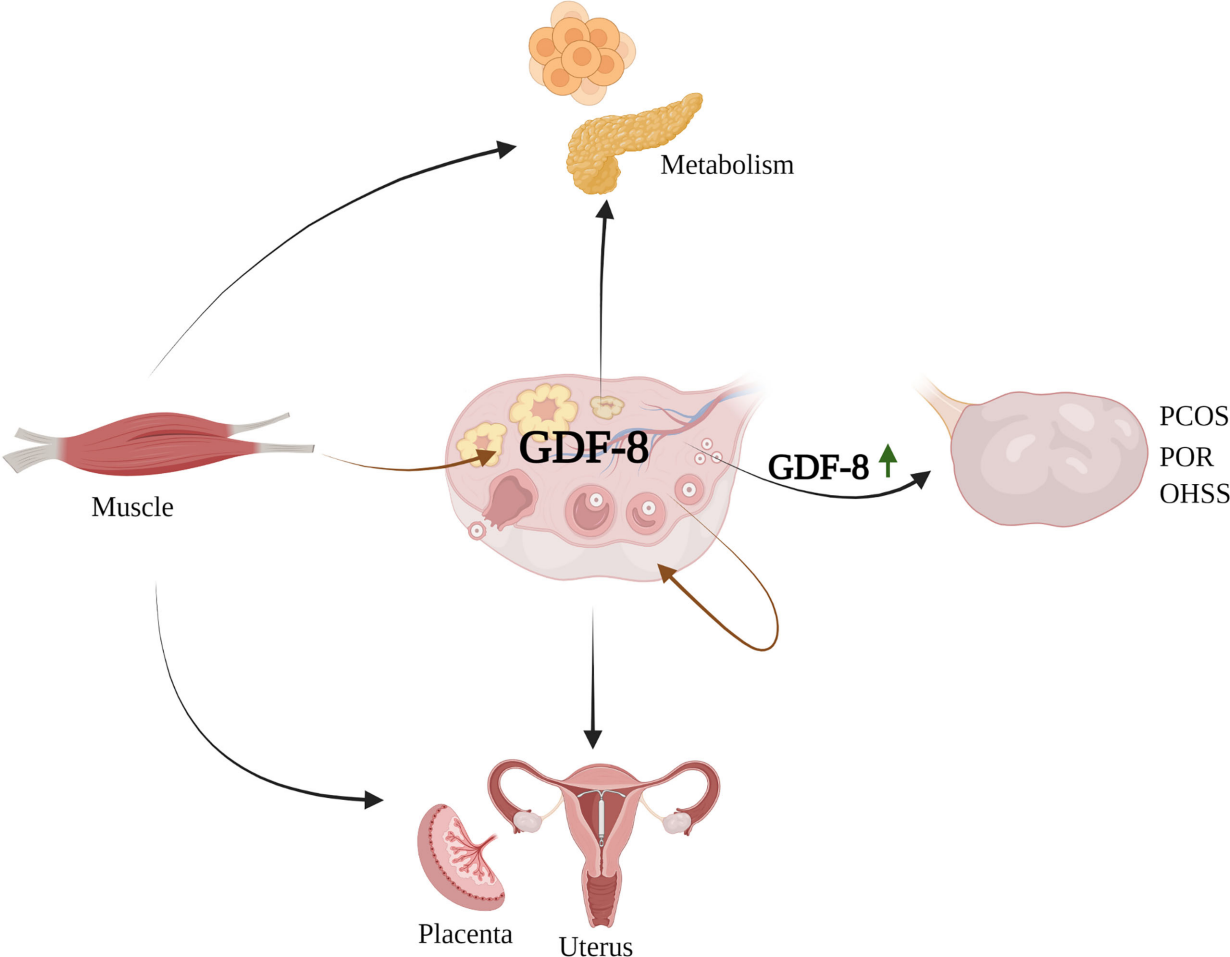
Secretion, distribution and effects of GDF-8 *in vivo.* PCOS, polycystic ovary syndrome; POR, poor ovarian response; OHSS, Ovarian hyperstimulation syndrome.

Herein, we describe the regulatory role and potential importance of GDF-8 in ovarian reproductive activity: GDF-8 expression *in vivo*; the role of GDF-8 in regulating reproductive functions in an *in vitro* GC model; association between GDF-8 expression and PCOS; relationship between GDF-8 expression and the ovarian response, pregnancy outcome of infertile women undergoing *in vitro* fertilization (IVF) procedures (**Scheme 1**). We also discuss the signaling pathways of the cellular responses to GDF-8, which could provide targets for alternative approaches in clinical treatment of ovarian reproductive disorders.

## Expression of GDF-8 and its Functional Receptors in the Human Ovary

In 2015, Chang et al. (36) demonstrated mature GDF-8 proteins to be detectable in the follicular fluid and GCs obtained from 16 infertile women undergoing IVF procedures on oocyte pick-up (OPU) day by enzyme-linked immunosorbent assay (ELISA). The GDF-8 concentration in follicular fluid was 2.01– 4.17 (mean ± SD, 2.95 ± 0.54) ng/mL. Those data were supported by four subsequent studies (37–40). The studies showed that the concentration range of GDF-8 in the follicular fluid of 102 IVF patients on OPU day was 1.00 – 8.00 ng/mL (**Table 1**). Notably, those study participants did not include women with PCOS or women with a poor-ovarian response (POR) or hyper-ovarian response (HOR) upon IVF. In addition, GDF-8 mRNA was detected in all three types of GCs (SVOG, KGN, and human granulosa-lutein (hGL) cells) (36, 39) and GDF-8 proteins were located in the cytoplasm and membrane of hGL cells (44). Those data suggested GCs to be a potential source of follicular GDF-8 protein.

**Table 1:** GDF-8 expression in human serum and follicular fluid.

Year	Women		Number of patients	Expression(mean ± SD, ng/mL)	Source	Time when samples were collected	Ref.
2012	Healthy volunteers		38	14.2 ± 9.7	Serum	For women with spontaneous menstrual cycles, blood samples were collected between day 3 and day 7 of a spontaneous menstrual cycle. Women with amenorrhea >3 months were asked to measure their basal body temperature, and blood samples were collected during the next cycle about 2–3 weeks following menstruation which occurred after spontaneous ovulation.	(41)
	PCOS	Non-obese	135	16.0 ± 6.4	Serum		
		Obese	104	17.4 ± 8.3	Serum		
		Total	239	16.6 ± 15.6	Serum		
2015	IVF patients		16	2.95 ± 0.54	FF	OPU day	(36)
2015	IVF patients		12	1.84 ± 0.82	FF	OPU day	(37)
2016	IVF patients	Pregnant	11	4.1 ± 0.4	Serum	hCG day	(42)
				4.0 ± 0.6	Serum	14 days after ET	
				1.6 ± 0.3	FF	OPU day	
		Non-pregnant	8	3.0 ± 0.4	Serum	hCG day	
				5.9 ± 0.9	Serum	14 days after ET	
				0.9 ± 0.2	FF	OPU day	
2019	Healthy volunteers	Non-obese	42	28.27 ± 35.48	Serum	Second day of early follicular phase of spontaneous bleeding	(43)
	PCOS	Non-obese	46	17.52 ± 11.20	Serum		
2020	IVF patients	Non-PCOS	40	4.71 ± 0.31	Serum	Gonadotropin administration	(38)
				4.37 ± 0.63	Serum	14 days after ET	
				1.65 ± 0.15	FF	OPU day	
		PCOS	40	7.15 ± 0.86	Serum	Gonadotropin administration	
				5.92 ± 0.66	Serum	14 days after ET	
				2.82 ± 0.21	FF	OPU day	
2021	IVF patients	Healthy	30	1.050 ± 0.130	FF	OPU day	(39)
		PCOS	29	2.220 ± 0.203	FF	OPU day	
2021	IVF patients	POR	20	3.14 ± 0.686	Serum	OPU day	(40)
				1.83 ± 0.389	FF	OPU day	
		HOR	20	2.42 ± 0.407	Serum	OPU day	
				1.34 ± 0.351	FF	OPU day	
		HOR	20	1.95 ± 0.451	Serum	OPU day	
				1.37 ± 0.359	FF	OPU day	

PCOS, polycystic ovary syndrome; IVF, in vitro fertilization; OPU, oocyte pick-up; ET, embryo transfer; FF, follicular fluid; hCG, human chorionic gonadotropin.

Lin et al. (33) measured expression of GDF-8 and protein expression of its receptors ACVR2A, ACVR2B and ALK5 by immunohistochemical staining in 34 women with normal ovaries. GDF-8 and its receptors were distributed within the growing follicles, surrounding stroma cells and corpus luteum. Specifically, immunostaining revealed GDF-8 and its receptors to be in the oocytes of primordial follicles as well as in the oocytes of ovarian follicles regardless of the developmental stage (antral follicles of diameter <10 mm). GDF-8 and its receptors (ACVR2A、ACVR2B、ALK5) were also localized in the GCs of primary antral follicles of diameter 0–2 mm, 2–5 mm and 5–10 mm, as well as large and small luteal cells of the corpus luteum in normal ovaries, respectively. Weak immunostaining of GDF-8 was detected in the antral space of several antral follicles. Expression of GDF-8 and its receptors appeared to increase progressively as the antral follicles matured. GCs displayed higher expression of GDF-8 and its receptors in antral follicles of diameter 2–5 mm and 5–10 mm. Compared with matched TCs, expression of GDF-8 and its receptors was significantly higher in GCs. Large luteal cells displayed higher expression of GDF-8 and its receptors in the corpus luteum. GDF-8 proteins were rarely observed in cells on the ovarian surface in their study.

**Figure 1:**
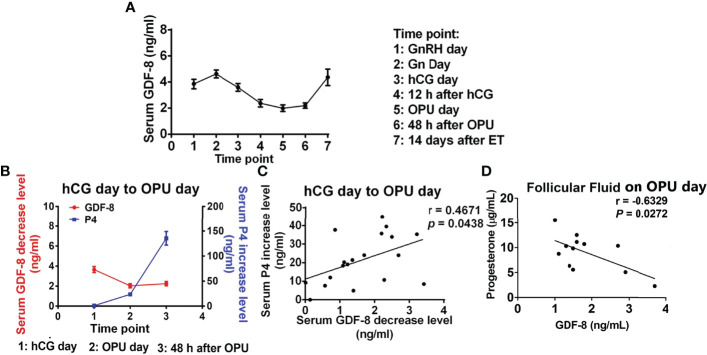
GDF-8 expression in serum during COH **(A)** and the relationship between the GDF-8 level and progesterone level in serum **(B, C)** and follicular fluid **(D)**. P4: progesterone. Reprinted with permission from references (37, 42).

The serum concentration of GDF-8 is higher than that in follicular fluid because GDF-8 produced in skeletal muscle, intra-follicular tissue, adipose tissue and cardiomyocytes contributes to the total serum concentration. It has been indicated that the GDF-8 concentration in human serum is 3 – 40 ng/mL (**Table 1**) (38, 40–43). Interestingly, Fang et al. (42) found the serum concentration of GDF-8 to change dynamically in patients undergoing controlled ovarian hyperstimulation (COH) during IVF treatment (**Figure 1A**). Blood samples from 19 patients undergoing COH were assayed at seven time points: GnRH-a day; Gonadotropin (Gn) day; human chorionic gonadotropin (hCG) day; 12 h after hCG administration; OPU day; 48 h after OPU day; and 14 days after embryo transfer (ET). The expression of GDF-8 in serum increased slightly after GnRH-a administration, but it decreased greatly after Gn injection, especially at 12 h after hCG administration, and increased in the late luteal phase.

GDF-8 may have an inhibitory role in regulation of progesterone production in the human ovary. Data from two studies (37, 42). involving 31 infertile women undergoing IVF procedures indicated GDF-8 expression to be negatively correlated with the progesterone level in serum and follicular fluid. The reduction in GDF-8 level was a positive correlation with the increase in the serum level of progesterone from hCG day to OPU day (**Figures 1B, C**). Furthermore, the concentration of GDF-8 and progesterone in follicular-fluid samples from 12 IVF patients suggested GDF-8 expression to be negatively correlated with the progesterone level in human follicular fluid on OPU day (37) (**Figure 1D**).

## Functional Roles of GDF-8 in the Regulation of Ovarian Functions

### GDF-8 Increases Estrogen Production and Decreases Progesterone Production

Steroid hormones produced by the ovary are key components in maintaining the female phenotype as well as supporting normal ovarian function, including follicular development, oocyte maturation, ovulation and embryo implantation. A hormone imbalance may negatively affect ovarian processes, thereby leading to adverse outcomes such as estrogen deficiency, ovulation disorders, premature ovarian failure and infertility. Therefore, optimal regulation of ovarian steroid production is imperative for female reproductive health.

Estrogen is one of the representative ovarian steroids that, together with Gns, promotes dominant follicular development and oocyte maturation by enhancing the growth and differentiation of GCs (45, 46). Estrogen is obtained from androgens catalyzed by aromatase for use within the ovary as well as for endocrine signaling to a host of tissues, including the uterus, breast, brain, bone, vascular system and skin appendages (47). In women of reproductive age, aromatase is a key enzyme for estrogen formation in the ovary, and GCs are important sites of estrogen formation (48).

Recent studies have revealed that GDF-8 increased aromatase/estradiol production in primary hGL cells (37, 49–51). Treatment of hGL cells obtained from IVF patients with GDF-8 (1, 10, or 100 ng/mL) for 24 h increased mRNA (*CYP19A1*) and protein expression of aromatase in a concentration-dependent manner. Significant stimulatory effects were obtained after treating cells with GDF-8 at 30 or 100 ng/mL. Treatment of hGL cells with GDF-8 (1, 10, or 100 ng/mL) for 48 h increased estradiol accumulation. Two studies (50, 51) revealed that GDF-8 can promote aromatase expression in a time-dependent manner in hGL cells; mRNA and protein expression of aromatase were increased significantly after GDF-8 (100 ng/mL) treatment for 6, 12, or 24 h. Those results suggest that GDF-8 may upregulate aromatase expression in a time- and dose-dependent manner, and then increase estradiol production.

Progesterone is an important steroid hormone and is secreted by hGL cells and luteal cells (which is differentiated from GCs) in the ovary. Aberrant secretion of progesterone in the ovary can lead to luteal-phase deficiency and premature luteinization (52, 53). The premature increase in the progesterone level before ovulation can have a negative impact on maturation of endometrium, which further affects synchronization between embryonic development and endometrial receptivity. After ovulation, maintaining high levels of progesterone is crucial for early implantation of the embryo. Therefore, precise modulation of ovarian progesterone secretion is required to maintain normal reproductive functions and to improve the chance of embryo implantation and the probability of pregnancy.

Progesterone is produced in three serial steps as follows; 1) Steroidogenic acute regulatory protein (StAR) transports cholesterol from the outer to the inner mitochondrial membrane, 2) cholesterol is converted to pregnenolone by cytochrome P450 cholesterol side-chain cleavage (P450scc) enzyme, and 3) 3β-hydroxysteroid dehydrogenase/Δ5-Δ4 isomerase (3β-HSD) converts pregnenolone to progesterone (54–56). StAR has been identified as the key regulatory protein that mediates cholesterol transfer and enhances the metabolism of cholesterol into progesterone (57, 58). It increases the flow of cholesterol into mitochondria, thus regulating substrate availability to whatever amount of P450scc is available.

GDF-8 can reduce progesterone production by downregulating StAR expression. Treatment of hGL cells with GDF-8 (1, 10, or 100 ng/mL) for 24 h reduces mRNA (*STAR*) and protein expression of StAR and progesterone accumulation in a concentration-dependent manner (38, 49). Significant effects have been observed when cells are treated with higher concentrations of GDF-8 (10 or 30 ng/mL). Fang and colleagues (37) showed that when SVOG cells were treated with GDF-8 (30 ng/mL) for 3, 12 or 24 h, GDF-8 initially downregulated mRNA (*STAR*) expression of StAR at 12 h, and the suppressive effect persisted until at least 24 h after treatment. In two studies (37, 50), treatment of SVOG cells or hGL cells with GDF-8 (30 ng/mL) did not affect mRNA expression of 3β-HSD (*HSD3B*) or P450scc enzyme (*CYP11A1*) at any time point examined.

### GDF-8 Regulates Gn Responsiveness

The pituitary gland-derived Gns FSH and LH are key players in germ-cell formation. Several essential processes for oocyte formation, such as stimulation of GC proliferation, estradiol production, antrum formation in secondary ovarian follicles, growth and maturation of antral follicles, are triggered by FSH and LH. FSH and LH, together, lead to folliculogenesis, oogenesis, meiotic maturation of oocytes and oocyte competence (59, 60). FSH and LH exert their effects through specific receptors (61, 62): the FSH receptor (FSHR) and LH receptor (LHR). By virtue of the FSHR and LHR, GCs become more competent in response to FSH and LH surge.

Chang and collaborators (49) revealed that treatment of hGL cells with GDF-8 (1, 10, or 100 ng/mL) for 24 h increased expression of FSHR mRNA and decreased expression of LHR mRNA in a concentration-dependent manner. Pretreatment with GDF-8 (30 ng/mL) enhanced FSH-induced expression of aromatase mRNA and protein significantly, and the LH-induced increase in progesterone production was abolished by pretreatment with GDF-8, as FSH or LH (0.2 IU/mL) was added to the hGL cell culture and incubation allowed for 24 h. Those data suggested that GDF-8 enhanced GC responsiveness to FSH by upregulating FSHR expression, thereby leading to significant enhancement of FSH-induced mRNA and protein expression of aromatase. Similarly, GDF-8 suppresses GC responsiveness to LH by downregulating LHR expression.

### GDF-8 Modulates Expansion of the Cumulus Oophorus

The occurrence of Cumulus oocyte complex (COC) and extent of expansion of the cumulus oophorus have been linked to oocyte competence. Animal models of disruption to the cumulus matrix frequently show impaired ovulation, which demonstrates that expansion of the cumulus oophorus is a critical ovulatory event (63). The extent of expansion of the cumulus oophorus is used as a criterion for oocyte selection in IVF procedures. Pentraxin 3 (PTX3) plays a key part in the process of assembling extracellular matrix (ECM), which is essential for expansion of the cumulus oophorus, ovulation and *in vivo* fertilization (64). A study in knockout mice has shown that depletion of PTX3 leads to infertility, featuring severe abnormalities in cumulus-oophorus expansion and failure of *in vivo* fertilization (65). Clinical data have revealed that PTX3 expression in cumulus cells (a specific sub-lineage of GCs) is positively correlated with the fertilization rate and quality of the corresponding oocytes (66, 67).

GDF-8 may modulate expansion of the cumulus oophorus by downregulating expression of the key linking protein (PTX3) in human GCs. In one study (36), treatment of SVOG, hGL and KGN cells with GDF-8 (1, 10, or 100 ng/mL) for 6 h downregulated mRNA and protein expression of PTX3 in a concentration-dependent manner. Knockdown of ALK5 expression reversed GDF-8 (30 ng/mL)-induced downregulation of mRNA expression of PTX3.

### GDF-8 Inhibits GC Proliferation

GCs have a major role in the development and maturation of oocytes *in vivo* and *in vitro*. GCs interact with oocytes and create a highly specialized microenvironment through the release of steroidal hormones and growth factors (68, 69). GCs are involved in a broad range of cellular physiological processes during follicular development, including mechanical support, nutrient intake, TC differentiation and steroidogenesis (68, 70). Any alterations in the metabolism, proliferation, differentiation and apoptosis of GCs may have a negative impact on the quality of the oocyte and resulting embryo.

Chang and coworkers (71) revealed that GDF-8 (30 ng/mL) added to SVOG cells every 24 h for 72 h suppressed cell proliferation significantly compared with that observed in the control group. GDF-8 (1, 10, or 100 ng/mL) reduced cell numbers in a concentration-dependent manner. GDF-8 (30 or 100 ng/mL) reduced the viability of SVOG cells significantly. GDF-8 could inhibit cell proliferation by upregulating connective tissue growth factor (CTGF) expression in GCs. This observation is based on two main phenomena. First, GDF-8 and CTGF suppressed proliferation of SVOG cells significantly. small interfering RNA (siRNA)-based strategies were used to show that GDF-8-induced inhibition of cell proliferation was mediated by CTGF and that targeted depletion of CTGF reversed the suppressive effect induced by GDF-8. The increase in CTGF expression contributed to the GDF-8-induced suppression of GC proliferation. Second, using dual-inhibition approaches (the inhibitor SB431542 and siRNAs), GDF-8 was found to induce upregulation of CTGF expression. The detailed molecular determinants of the signaling mediating the GDF-8 induced up-regulation of CTGF have not been elucidated.

### GDF-8 and ECM Formation

The ECM within a follicle plays a critical part in providing structural support, promoting oocyte maturation, restricting access of growth factors and hormones to the follicle and influencing various cellular processes (morphology, communication, proliferation, aggregation, survival and steroidogenesis) (72). Lysyl oxidase (LOX) is an extracellular enzyme essential for ECM stabilization. LOX catalyzes the final step of the crosslinking of collagen and elastin, which are two major components of a mature and functional ECM (73, 74). In rats, LOX expression in mural GCs was found to correlate with the developmental competence of oocytes, indicating that LOX may serve as a biomarker of oocyte quality for assisted reproduction (75).

It was shown that treatment of SVOG cells and non-immortalized primary hGL cells with GDF-8 (1, 10, or 100 ng/mL) increased mRNA (12 h) or protein (24 h) expression of LOX in a concentration-dependent manner. Time-course study following GDF-8 (30 ng/mL) treatment demonstrated that mRNA expression of LOX began to increase 6 h after treatment, and persisted until at least 24 h, whereas protein expression of LOX began to increase at 12 h (76).

### GDF-8 Impairs Glucose Metabolism

Analyses of glucose uptake showed that insulin-induced glucose uptake was inhibited by GDF-8 (30 ng/mL) in hGL cells (39). Measurement of lactate accumulation showed that GDF-8 abolished insulin-induced lactate production, and did not affect the expression of insulin signaling pathway-related proteins, including insulin receptor substrate-1 (IRS-1), IRS-2, insulin receptor and glucosetransporter 4. GDF-8 binds to ALK5 receptor and increases production of serine protease inhibitor family E member 1, and then impairs the glucose metabolism of GCs. Impaired glucose metabolism may reduce the energy supply to GCs and oocytes and, thus, compromises GC proliferation and oocyte development (77).

### GDF-8 Signaling

**Figure 2:**
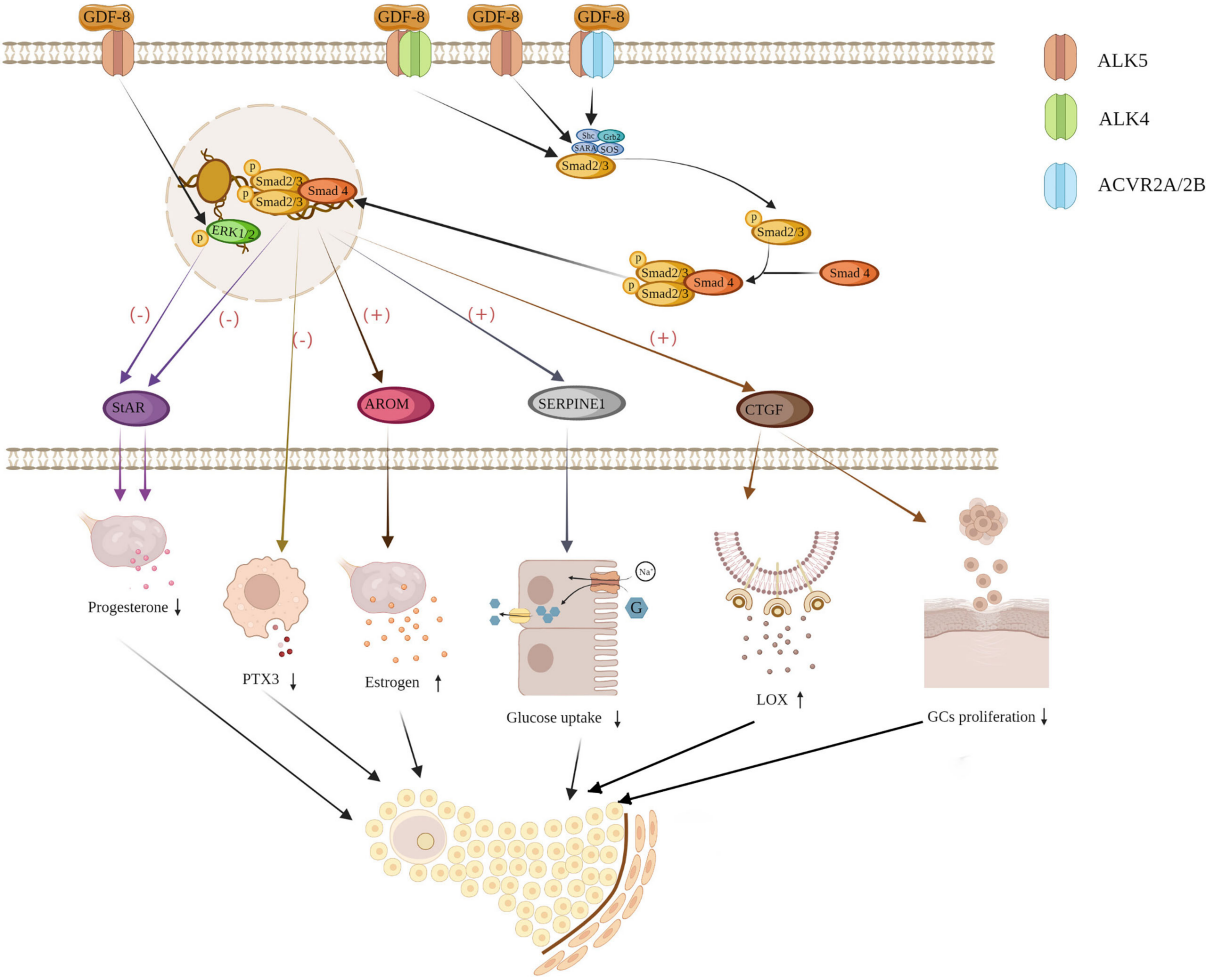
Proposed molecular mechanism of GDF-induced regulation in GCs (schematic). ALK5, ALK4 and ACVR2A/2B, GDF-8 receptors; AROM, aromatase; CTGF, connective tissue growth factor; LOX, lysyl oxidase; PTX3, pentraxin 3; StAR, steroidogenic acute regulatory protein; SERPINE1, serine protease inhibitor family E member1.

Many studies have revealed that the ALK5-mediated SMAD2/3-SMAD4 signaling pathway is indispensable for GDF-8-induced regulation of protein expression and accumulation in human GCs (**Figure 2**). GDF-8 upregulates aromatase expression by activating the ALK5-mediated SMAD2/3 signaling pathway (49), and suppresses progesterone production through ALK5-mediated SMAD2/3 and extracellular signal-regulated kinase 1/2 signaling pathways (37). The suppressive effect of GDF-8 on PTX3 is mediated by a complex comprising ALK5 and ACVR2A/ACVR2B, and involves SMAD-dependent signaling (36). GDF-8 induces upregulation of CTGF expression through ALK4/5-mediated SMAD2/3-dependent signaling pathways (71, 76). Notably, ALK5, but not ALK4, was found to be the primary physiological receptor for GDF-8 in human GCs in several studies (36, 37, 76). This is consistent with previous findings that GDF-8 utilizes ALK4 in myoblast cells, whereas in non-myogenic cells GDF-8 prefers the use of ALK5 (78).

**Figure 3:**
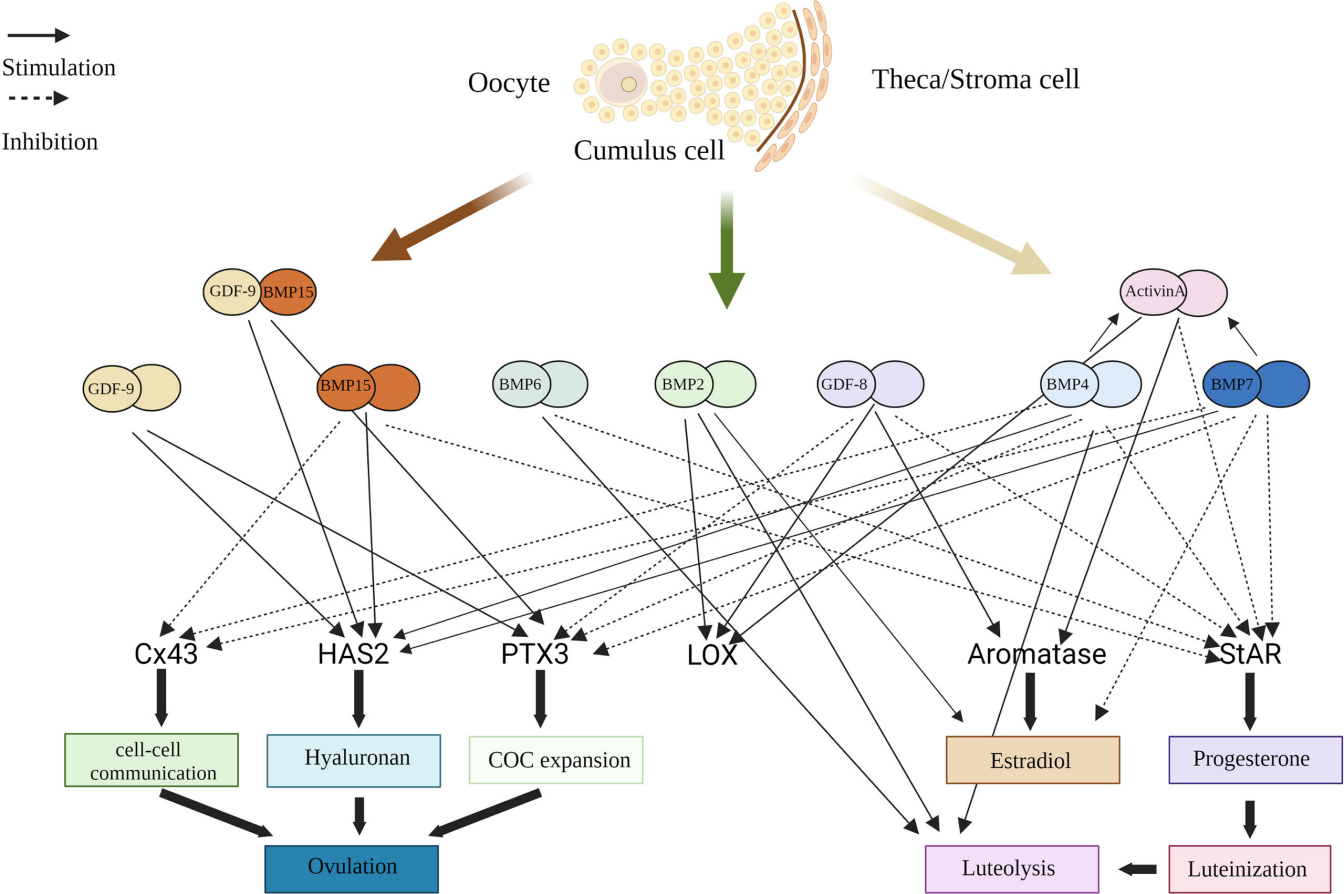
Schematic diagram summarizing functional roles of BMPs, GDF-8, GDF-9, and Activin in the human ovary. BMP, bone morphogenetic protein; COC, cumulus oophorus complex; Cx43, connexin 43; GC, granulosa cell; HAS2, hyaluronan synthase type 2; PTX3, pentraxin 3; StAR, steroidogenic acute regulatory protein; LOX, lysyl oxidase.

Growth factors belonging to the TGF-β superfamily act on different signaling pathways in GCs to produce similar effects in human GCs (**Figure 3**). Studies have shown that in human GCs, progesterone is downregulated by activin *via* ALK4-SMAD2/3-SMAD4 (79, 80), BMP4 and BMP7 *via* ALK3-SMAD1/5/8-SMAD4 (81), BMP-9 *via* ALK1/2-SMAD1/5/8 and ALK5-SMAD2/3-SMAD4 (10), BMP15 *via* ALK3-mediated SMAD1/5/8 signaling pathway (82). Expression of PTX3 in GCs is influenced by activin binding to ALK4 (83), BMP2 binding to ALK2/3-BMPR2/ACVR2A inducing SMAD2/3-SMAD4 pathway (84), and BMP4 binding to ALK3/ALK6, BMP7 binding to ALK2/ALK3 inducing SMAD1/5/8 phosphorylation (85). The expression and activity of LOX are upregulated by activin A binding BMP type I receptor (86), and BMP2 binding activin/TGF-β type I receptor (87). AMH transduces its effects through AMH receptor type 2 and ALK2/3/6, and the SMAD1, SMAD5, or SMAD8 proteins (4).

The observation of different mechanisms of GDF-8 signaling in GCs responses to growth factors and signaling in muscle cells and non-muscle cells is critical for the development of new pharmacological strategies for clinical citation, as differential strategies of signaling can be applied to minimize off-target effects. Pharmaceutical strategies have been used in several muscle and metabolic disorders, but these treatments have been clinically suboptimal for some reasons including potential clinical toxicities due to lack of target specificity (88–91).

To date, the available studies suggest that activins, GDFs and BMPs have a role in follicle development. GDF-8 is structurally similar to activin isoforms (92). In this context, GDF-8 may regulate steroid synthesis, follicle maturation and ovulation together with growth factors from the TGF-β superfamily in the human ovary. In brief, GDF-8 may have a functional role, likely by acting as a maturation stimulator and luteinization inhibitor to enhance differentiation of the dominant follicle and subsequent formation of the corpus luteum after ovulation.

## Aberrant Expression of GDF-8 and Female Reproductive Disorders

### GDF-8 and PCOS

PCOS is the most common endocrine disorder affecting women of reproductive age, with a reported prevalence of 8-13% (93–95). PCOS prevalence is increased (to >25%) among severely obese women, and comorbid obesity aggravates all PCOS symptoms (96). PCOS is accompanied by substantial metabolic abnormalities, including hyperinsulinemia, insulin resistance and dyslipidemia. Recent studies have shown the role of GDF-8 in regulating the metabolism of glucose and fat, which is associated with obesity, insulin resistance and diabetes mellitus development (97–99), which are also important features in PCOS women. Clinical studies have shown the possible involvement of GDF-8 in PCOS pathogenesis (38, 41, 43).

**Figure 4:**
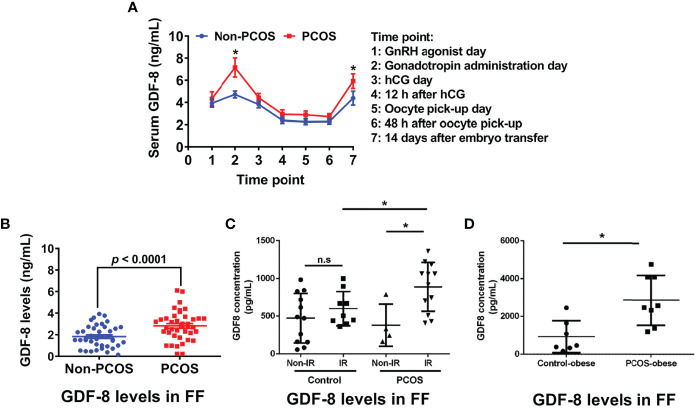
Expression of GDF-8 in the follicular fluid and serum of PCOS patients and non-PCOS patients. **(A, B)** GDF-8 expression at different time points during COH in serum **(A)** and GDF-8 expression in follicular fluid **(B)** collected at OPU day measured by ELISA. **(C)** GDF-8 concentration in follicular fluid derived from control patients and PCOS patients with or without IR. **(D)** Comparison of GDF-8 expression in obese patients with or without PCOS. Results are the mean ± SEM. *P <0.05 non-PCOS group vs. PCOS group at an identical time point. n.s., no significant difference; FF, follicular fluid; IR, insulin resistance. Reprinted with permission from references (38, 39).

GDF-8 expression in serum is higher in women with PCOS than that in women not suffering from PCOS (41). Notably, high expression of GDF-8 is found only in obese women with PCOS, whereas there is no difference between nonobese women regardless of PCOS status (**Table 1**) (41, 43). GDF-8 expression in the serum of women with PCOS is positively associated with obesity-related variables such as the fasting glucose level, body mass index (BMI), and waist circumference. GDF-8 expression in serum also changes dynamically in women with PCOS undergoing COH during IVF treatment (**Figure 4A**). Fang and colleagues (38) showed that, compared with women not suffering from PCOS, the serum GDF-8 concentration of PCOS patients was higher on GnRH-a day, Gn day, hCG day, 12 h after hCG administration, OPU day, 48 h after OPU and 14 days after ET. In particular, the differences were significant on Gn day and 14 days after ET.

The change in GDF-8 expression in follicular fluid is similar to that in the serum of PCOS patients. GDF-8 expression in follicular-fluid samples obtained from women undergoing oocyte retrieval during IVF treatment was examined (**Table 1**). GDF-8 expression was significantly higher in the follicular fluid of PCOS patients than in that of non-PCOS patients (38, 39) on OPU day (**Figure 4B**). Bai et al. (39) measured GDF-8 expression in follicular fluid stratified by homeostatic model assessment of insulin resistance. GDF-8 expression was significantly higher in women with PCOS and IR than that in women with PCOS without IR, whereas there was no difference between women with IR and women without IR in the study’s non-PCOS group (**Figure 4C**). GDF-8 expression was also higher in obese women with PCOS than that in obese women not suffering from PCOS (**Figure 4D**) and the GDF-8 concentration was positively correlated with BMI (39).

High expression of GDF-8 in the follicular fluid of women with PCOS can be attributed (at least in part) to aberrant expression of GDF-8 in human GCs. In one study, expression of GDF-8 mRNA was measured in hGL cells collected from 30 PCOS and 29 non-PCOS patients using quantitative polymerase chain reaction (qPCR) (39). qPCR results showed that expression of GDF-8 mRNA was higher in the PCOS group than that in the non-PCOS group. Those data were consistent with results from another study (33) that detected (using immunohistochemical methods) significantly increased expression of GDF-8 proteins in the ovarian GCs of PCOS patients. In 14 ovary samples from women with PCOS, staining of GDF-8 and ALK5 was strongly detected in GCs and TCs in antral follicles of various diameters (0–2 mm, 2–5 mm, and 5–10 mm).

The results of clinical studies combined with the results of *in vitro* studies on GCs suggested that the elevated GDF-8 in serum and follicular fluid in women with PCOS inhibited PTX3 expression and granulosa cell proliferation, which might lead to failed cumulus expansion and subsequent ovulatory dysfunction, as well as inhibition and glucose metabolism leading to abnormal glucose metabolism.

### GDF-8 and POR

COH with Gns is an essential step in assisted IVF. Conventional COH approaches lead to sufficient follicular growth and an appropriate estrogen level in most women. The number of mature oocytes retrieved is the parameter most commonly used to assess the ovarian response to COH, as the oocyte number is closely correlated with the likelihood of achieving a live birth in IVF (100, 101). Based on the oocyte number, the effect of COH reliant on the ovarian response is divided into POR, normal ovarian response (NOR) and HOR clinically (102–104). POR and HOR are pathologic conditions in COH. Multiple factors have been reported to act in parallel with regard to the ovarian response. The balance of the follicular microenvironment would be disrupted by aberrant expression of growth factors in follicles. And then, the ovarian response would be impaired subsequently.

Bai and colleagues (40) showed that the follicular-fluid and serum concentrations of GDF-8 were significantly higher in POR patients than those in NOR patients, with no significant differences in such concentrations between NOR and HOR patients on OPU day. The data were from 60 non-PCOS women who underwent COH for male factors or fallopian-tube factors, with 20-women each in the POR, HOR and HOR groups (**Table 1**). Protein expression of GDF-8 and ALK5 in GCs was higher in POR patients than that in NOR patients, whereas there was no significant difference in such expression between HOR and NOR groups. Correlation analysis showed that on OPU day, GDF-8 expression was negatively correlated with the LH level, antral-follicle count and estradiol level, and GDF-8 expression did not correlate with FSH or AMH concentrations. The result was not in accordance with the positive correlation documented between GDF-8 expression and estrogen level in cell experiments, which might be due to the fact that estradiol is also produced in several organs *in vivo*, including the adrenal glands, brain, adipose tissue, skin, pancreas (47), and other sites yet to be identified.

### GDF-8 and Ovarian Hyperstimulation Syndrome (OHSS)

OHSS is a serious complication associated with COH. OHSS is a potentially iatrogenic condition that can result in massive ovarian enlargement, hydrothorax, ascites, acute respiratory distress syndrome, renal failure- and, rarely, death (estimated at 3 per 100,000 population) (105, 106). GDF-8 has been reported to the associated with OHSS development (51). GDF-8 expression in the follicular fluid of 25 OHSS patients was found to be upregulated compared with that of 25 control patients. Moreover, mRNA expression of GDF-8 was higher in the hGL cells of OHSS patients than in those of control patients. However, mRNA expression of ALK5 in hGL cells did not vary significantly between control cases and OHSS patients in that study.

## diagnostic approaches and Potential therapeutic

### Diagnostic Approaches

Serum levels of hormones (especially estradiol and progesterone) vary during COH and influence pregnancy outcome for patients treated with IVF-ET (53, 107, 108). According to the studies mentioned above (37, 38, 49–51), cell experiments and clinical data showed GDF-8 expression to be related to levels of estrogen and progesterone. Therefore, studies focused on the relationship between GDF-8 expression in serum and pregnancy outcomes in IVF-ET patients were subsequently carried out (38, 42). GDF-8 expression in serum was found to be a valuable predictor for pregnancy for patients treated with IVF-ET. The studies concluded that before hCG administration, higher expression of GDF-8 may be beneficial for pregnancy due to maintenance of a lower progesterone level in serum. After hCG administration, lower expression of GDF-8 may be crucial for early implantation of the embryo due to maintenance of a high level of progesterone.

**Figure 5:**
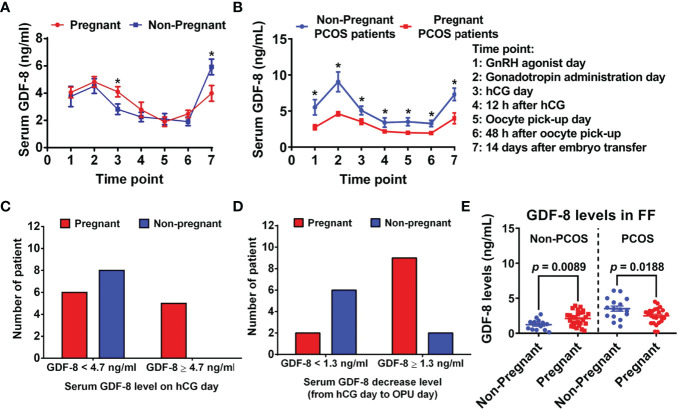
Relationship between GDF-8 expression and pregnancy outcome. **(A)** GDF-8 expression in serum at different time points in non-pregnant and pregnant non-PCOS patients (11 pregnant women and 8 non-pregnant women). **(B)** GDF-8 expression in the serum of pregnant and non-pregnant PCOS patients (24 pregnant women and 16 non-pregnant women). **(C)** Relationship between GDF-8 expression in serum with pregnancy outcome on hCG day of two groups according to a cutoff GDF-8 value in serum on hCG day (GDF-8 <4.7 ng/mL *vs.* GDF-8 ≥4.7 ng/mL). **(D)** Relationship between GDF-8 expression in serum with pregnancy outcome from hCG day to OPU day of two groups according to the cutoff value for a decrease in GDF-8 expression in serum from hCG day to OPU day (GDF-8 decrease <1.3 ng/mL *vs.* GDF-8 decrease ≥1.3 ng/mL). **(E)** GDF-8 expression in the follicular fluid of non-pregnant and pregnant patients. Pregnant group = red and non-pregnant group = blue. *P<0.05 compared between the non-pregnant group and the pregnant group at the same time point. FF, follicular fluid. Reprinted with permission from references (38, 42).

Specifically in one study (42), 19 COH patients (not including PCOS patients) were divided into a pregnant group (n = 11) and non-pregnant group (n = 8) based on pregnancy outcome. Variation in GDF-8 expression in serum was similar in both groups (**Figure 5A**) at seven time points: GnRH-a day; Gn day; hCG day; 12 h after hCG administration; OPU day; 48 h after OPU; 14 days after ET. There were significant differences in GDF-8 expression between the pregnant group and the non-pregnant group on hCG day and at 14 days after ET. On hCG day, GDF-8 expression in serum was significantly higher in the pregnant group than in the non-pregnant group (4.1 ± 0.4 *vs.* 3.0 ± 0.4, *p* = 0.038). However, GDF-8 expression in serum 14 days after ET was significantly lower in the pregnant group than in the non-pregnant group (4.0 ± 0.6 *vs.* 5.9 ± 0.9, *p* = 0.048). Reductions in serum GDF-8 expression from hCG day to OPU day were more pronounced in the pregnant group than in the non-pregnant group (1.9 ± 0.2 *vs*. 1.1 ± 0.4, *p* = 0.044). In addition, GDF-8 expression in follicular fluid collected on OPU day was significantly higher in the pregnant group than in the non-pregnant group (1.6 ± 0.3 *vs.* 0.9 ± 0.2, *p* = 0.039).

The value of GDF-8 to predict pregnancy outcome was further analyzed in 19 COH patients (42). GDF-8 expression in serum on hCG day and a decrease in GDF-8 expression from hCG day to OPU day were pivotal predictors for pregnancy outcome. Patients with GDF-8 expression >4.7 ng/mL had a higher pregnancy prevalence on hCG day (**Figure 5C**). From hCG day to OPU day, patients with a decrease in GDF-8 expression >1.3 ng/mL had a higher pregnancy prevalence (**Figure 5D**).

Fang and collaborators (38) studied the relationship between GDF-8 expression and pregnancy outcome in 40 PCOS patients. GDF-8 expression of pregnant PCOS patients and non-pregnant PCOS patients were compared at seven time points during COH. GDF-8 expression in serum was significantly lower in the pregnant group than in the non-pregnant group during COH (**Figure 5B**), and follicular GDF-8 expression was lower in pregnant PCOS patients than that in non-pregnant PCOS patients on OPU day (**Figure 5E**). Those results suggest that high GDF-8 expression may negatively affect pregnancy outcome in IVF patients with PCOS.

The relationship between GDF-8 expression and pregnancy outcome in non-PCOS patients and PCOS patients suggests that GDF-8 expression should be regulated dynamically and accurately in a certain range that is beneficial to pregnancy. Although the predictive value of GDF-8 expression for pregnancy outcome has been revealed, the underlying mechanism is not known. It is worth noting that GDF-8 expression is negatively correlated with the progesterone level *in vivo*, which plays an important part in ensuring successful pregnancy. Future investigation of the mechanism will be needed to support and guide new strategies for clinical treatment.

### Potential Therapeutic

**Figure 6:**
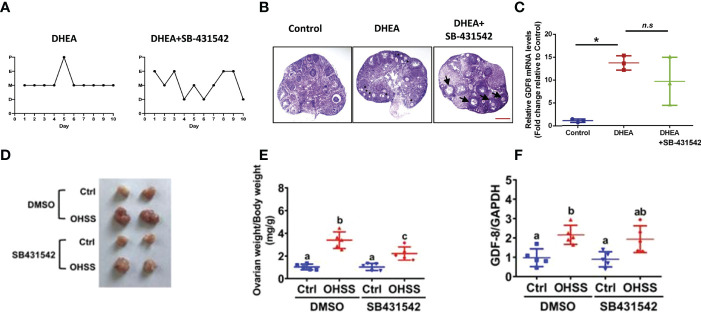
Effect of SB431542 in a mouse model of PCOS **(A–C)** and rat model of OHSS **(D–F)**. **(A)** Stage analysis of the estrus cycle of the SB431542-treatment group and control group in mice, **(B)** H&E staining of representative ovaries of mice in the SB431542-treatment group and control group. **(C)** Changes in GDF-8 expression in the SB431542-treatment group and control group. **(D)** Representative photograph of ovaries in rats in the control group and OHSS group. **(E)** Ovarian weight over bodyweight of rats in the control group and OHSS group. **(F)** Expression of GDF-8 mRNA in rat ovaries in the control group and OHSS group. Scale bar: 100 μm. Values without a common letter are significantly different (p < 0.05). Values without a common letter are significantly different (p < 0.05). Reprinted with permission from references (39, 51).

Recently, the results of animal studies suggested that GDF-8 receptor AKL5 inhibitors might be potential therapeutic agents for the treatment of OHSS and ovulation disorders in PCOS. Bai and collaborators (39) undertook a study based on dehydroepiandrosterone (DHEA)-induced PCOS in mice followed by treatment with SB431542 (specific inhibitor of ALK5). The estrus cycles in mice treated with SB431542 recovered partially (**Figure 6A**). Staining (hematoxylin and eosin) showed that SB431542 treatment improved DHEA-induced PCOS-like ovaries in mice significantly. The morphology of polycystic ovaries was alleviated, and several follicles could mature (**Figure 6B**). GDF-8 expression in the SB431542-treatment group tended to decrease, but not significantly (**Figure 6C**). In addition, SB431542 was applied in a rat model of OHSS to block the function of GDF-8 (51). SB431542 administration attenuated the increases in ovarian size and bodyweight in the OHSS group (**Figures 6D, E**). qPCR showed that the mRNA expression of aromatase was upregulated significantly in the OHSS group and this induction was attenuated by SB431542 administration. However, upregulation of GDF-8 mRNA expression was not affected by SB431542 administration (**Figure 6F**).

## Discussion

GDF-8 is present in the human ovary and is produced by GCs. Cellular and animal experiments, as well as clinical data, suggest that GDF-8 is involved in ovarian reproductive functions, and that GDF-8 expression should be limited to a certain range with dynamic regulation to maintain normal ovarian reproductive functions. Some ovarian-related disorders, such as PCOS, OHSS and POR, are characterized by increased GDF-8 expression in serum and follicular fluid.

The significant effects of GDF-8 on downstream proteins expression and steroid production were observed when GCs cells *in vitro* treated with GDF-8 concentrations exceeded 10.0 ng/mL. Currently, it was reported that the GDF-8 levels in the human follicular fluid ranged from 1.0 to 8.0 ng/mL. However, the exact concentration of GDF-8 in the microenvironment in GCs residing in the human ovary remains unknown. It has been suggested that the physiological concentration of GDF-8 in the microenvironment where GCs located may be higher than 8.0 ng/mL, even higher than 10.0 ng/mL (37). This suggestion was based on the following reasons:1. GDF-8 used in studies *in vitro* was rhGDF-8, which was derived from mouse myeloma cell line. The differences of the bioactivity between the recombinant protein and the native protein are still unknown; 2. GDF-8 has been shown to regulate ovarian functions through autocrine/paracrine mechanisms; 3. It was reported that the concentrations of GDF-8 in human serum were up to 40 ng/mL.

Follicular development, ovulation and luteal formation are complex processes throughout the human menstrual cycle that involve several neural, neuroendocrine, endocrine and paracrine/autocrine control systems. GCs-secreted activin A, oocyte-derived BMP15 and GDF9, which are prominent local factors associated with normal ovarian function and follicular development, have a synergistic or inhibitory effect on each other (2, 109). The biological action of BMP15 and GDF9 might be synergistic (110). The human BMP15/GDF9 heterodimer is approximately 1000- to 3000-fold more potent than the human BMP15 homodimer (111). GDF9 blocked the suppressive effects of activin A on progesterone synthesis by increasing the expression of inhibin B, which acts as an activin A competitor (112). Furthermore, the stimulatory effect of activin A on follicular growth was augmented by cotreatment with growth hormone and inhibited by Follistatin, which is a binding protein for activin and inhibin produced by granulosa cells and present in follicular fluid (113). Interactions between GDF-8 and other growth factors have not been reported. The paracrine actions of GDF-8 in the ovary should not be considered in isolation, but as part of a molecular network with many intricate relationships, which together with Gns, steroid hormones and multiple intraovarian growth factors affect ovarian reproductive function in a synergistic or inhibitory biological role.

Dysregulation of several growth factors is associated with PCOS pathology. Activin A expression is lower in the serum of women with PCOS than in normal women (114, 115), but not significantly different in the follicular fluid (116). The expression of BMP15 and GDF9 were reduced and delayed in the oocytes and GCs of follicles in PCOS ovaries during the early follicular stage (117), and per oocyte was higher among women with PCOS (118). The GDF9 expression in cumulus GCs from patients with PCOS was significantly lower than the normal (119). The concentrations of activin A, BMP15 and GDF9 proteins are higher in follicular fluid than in serum. Indeed, activin A, BMP15 and GDF9 concentrations are very low in serum (in the pg/mL range), leading to the quantitation in serum being difficult. A recent study showed that BMP15 and GDF9 were detectable in 61% and 29% of women (120). Serum AMH levels are significantly higher in women with PCOS than in normal ovulatory women (121, 122). The importance of serum AMH as a useful tool in the prediction of PCOS and primary ovarian failure has also been acknowledged (123). GDF-8 level in serum of obese PCOS women is significantly higher than in normal women and is readily detectable in the serum of PCOS patients. Current studies have shown that GDF-8 concentrations in serum are above 3.0 ng/ml, which is several orders of magnitude higher than activin A, BMP15 and GDF9 concentrations in serum. GDF-8 may be a diagnostic marker for PCOS as AMH. However, it should be taken into account that the GDF-8 proteins in serum contain the fraction of muscle cells secreted.

GDF-8 expression in serum and follicular fluid is positively correlated with BMI, so weight control may be a way to limit GDF-8 expression. Comprehensive understanding of the basic functions and signaling pathways of GDF-8 will benefit development of strategies to treat related disorders. Some studies have indicated that the ALK5 receptor mediates the GDF-8 signaling pathway, and that the ALK5 inhibitor SB431542 alleviates polycystic ovarian morphology and OHSS symptoms. Therefore, drugs targeting the GDF-8 signaling pathway (e.g., inhibitors of ALK5 receptors) may be promising candidates. ALK5 receptors have irreplaceable effects on cell reproductive capacity, growth, wound regeneration and immune response. ALK5 receptors are widely present in several organs and tissues, including the breast, colon, liver, stomach, ovary and cervical (124–126). Using ALK5-receptor inhibitors prepared as targeted nanoparticles is a better strategy to improve their distribution in the ovary and reduce systemic adverse effects.

The morphology and development of the reproductive system in GDF-8 knockout animals have not been analyzed or reported in detail in the currently available studies. Future studies aimed at addressing these questions regarding human ovarian tissue or animal models may contribute to a better understanding of female reproductive disorders. Meanwhile, the expression profile of GDF-8 in developing follicles, the role of GDF-8 in the molecular network, and the comparison of the linkage and effects with other regulatory factors are directions that need to be investigated in the future.

## Conclusions

Studies *in vitro* have demonstrated that GC proliferation, steroidogenesis, expansion of cumulus oophorus, glucose metabolism and oocyte maturation are regulated by GDF-8. Clinical data have shown that GDF-8 mRNA and proteins, and its receptor ALK5, are expressed widely in the human ovary. Clinical studies have revealed the dynamic changes in GDF-8 expression in serum during COH, and that GDF-8 expression in follicular fluid and serum is correlated with the ovarian response and pregnancy outcome during IVF. GDF-8 has been revealed to be a potential component in the pathogenesis of PCOS and OHSS *via* clinical research and animal experiments.

## Author Contributions

XLZ and YQZ conceptualized and wrote the manuscript. YY and YCZ contributed to the planning and design of the manuscript. XZ and DXQ provided clinical oversight and expertise. CHZ motivated the article and provided funds for open access. All authors edited, revised, and approved the final version of this review.

## Funding

This work was supported by the Public Welfare Technology Research Project of Zhejiang Province (LGF21H160023) and National Natural Science Foundation of China, General Program (81802587).

## Conflict of Interest

The authors declare that the research was conducted in the absence of any commercial or financial relationships that could be construed as a potential conflict of interest.

## Publisher’s Note

All claims expressed in this article are solely those of the authors and do not necessarily represent those of their affiliated organizations, or those of the publisher, the editors and the reviewers. Any product that may be evaluated in this article, or claim that may be made by its manufacturer, is not guaranteed or endorsed by the publisher.

## Abbreviations

GCs, Granulosa cells; TCs, Thecal cells; GnRH-a, gonadotropin-releasing hormone agonist; LH, Luteinizing hormone; FSH, Follicle-stimulating hormone; TGF-β, Transforming growth factor-beta; BMP, bone morphogenetic proteins; AMH, anti-Müllerian hormone; GDF, Growth differentiation factor; IR, Insulin resistance; ALK, activin receptor-like kinase; ACVR2, activin receptor type 2; SMAD, mothers against decapentaplegic homolog; Cx43, connexin 43; HAS2, hyaluronan synthase type 2; PCOS, Polycystic ovary syndrome; IVF, *In vitro* fertilization; OPU, Oocyte pick-up; ELISA, Enzyme-linked immunosorbent assay; POR, Poor ovarian response; HOR, Hyper ovarian response; hGL, human granulosa-lutein; COH, Controlled ovarian hyperstimulation; Gn, Gonadotropin; hCG, Human chorionic gonadotropin; ET, Embryo transfer; StAR, Steroidogenic acute regulatory protein; P450scc, P450 side-chain cleavage enzyme; 3β-HSD, 3β-hydroxysteroid dehydrogenase; FSHR, Follicle-stimulating hormone receptor; LHR, Luteinizing hormone receptor; COC, Cumulus oocyte complex; PTX3: Pentraxin 3; ECM, Extracellular matrix; CTGF, Connective tissue growth factor; siRNA, small interfering RNA; LOX, Lysyl oxidase; IRS-1, insulin receptor substrate-1; BMI, Body Mass Index; qPCR, Quantitative polymerase chain reaction; NOR, Normal ovarian response; OHSS, Ovarian hyperstimulation syndrome; DHEA, dehydroepiandrosterone.
